# Part I: Before you apply for that faculty position …

**DOI:** 10.1111/cns.14359

**Published:** 2023-07-14

**Authors:** Rehana K. Leak, Devika Soundara‐Manickam, Xiaoming Hu, Jun Chen, Khalid M. Kamal, Wilson S. Meng

**Affiliations:** ^1^ Graduate School of Pharmaceutical Sciences School of Pharmacy, Duquesne University Pittsburgh Pennsylvania USA; ^2^ Department of Neurology University of Pittsburgh School of Medicine Pittsburgh Pennsylvania USA; ^3^ Department of Pharmaceutical Systems and Policy School of Pharmacy, West Virginia University Morgantown West Virginia USA

**Keywords:** academia, career, interview, job talk, research, tenure

## 1 INTRODUCTION

The objective of this two‐part *CNSNT* editorial is to provide real‐world, frank advice for scientists joining an academic or research institution. Based on our experience on hiring committees and mentorship of the next generation of scientists, we offer help for colleagues seeking positions at R1 (very high research activity) and R2 (high research activity) institutions in the United States, as defined by the *Carnegie Classification of Institutions of Higher Education*, and the equivalent in other countries. Most of the information applies to scientists researching the health sciences, but a lot of the advice applies to *any* faculty position. We will help you gain insights into the demands of the new position and evaluate the strengths and weaknesses of your application—before you apply. We will also try to help you prepare for the interview, land the job offer, and negotiate the contract.

**FIGURE 1:**
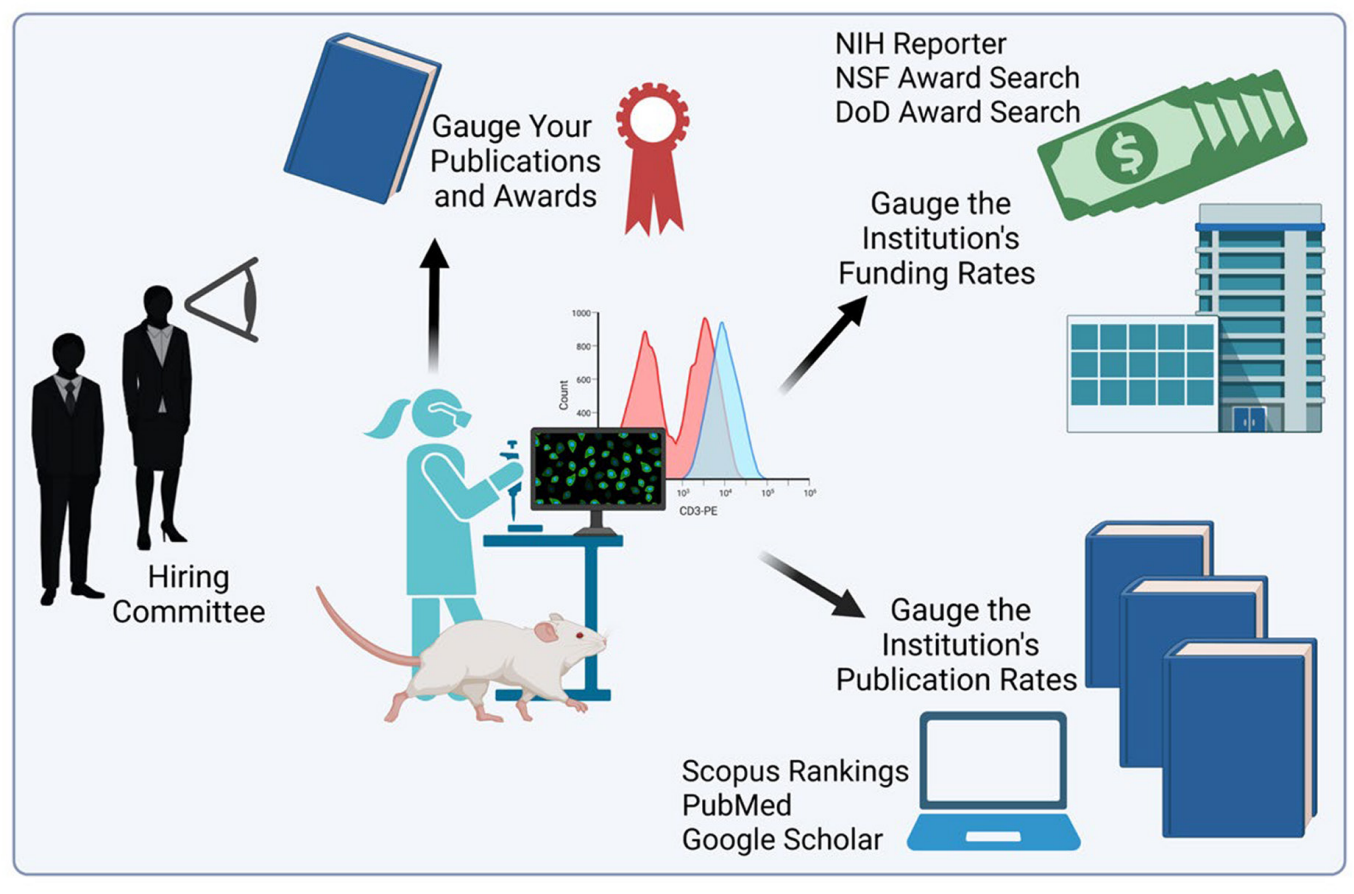
Planning ahead for a productive job interview. DoD, Department of Defense; NIH, National Institutes of Health; NSF, National Science Foundation. Created in BioRender®.

Before you spend the time applying, determine where the faculty members publish and how much extramural funding they bring to the institution (more on this below). Papers, citations, and externally funded grants are benchmarks against which your applications for retention, tenure, and/or promotion will be judged—now and in the future (Figure 1). You can request the faculty handbook (if not available online) and search all the criteria to achieve retention/tenure/promotion at that specific institution.

**FIGURE 2:**
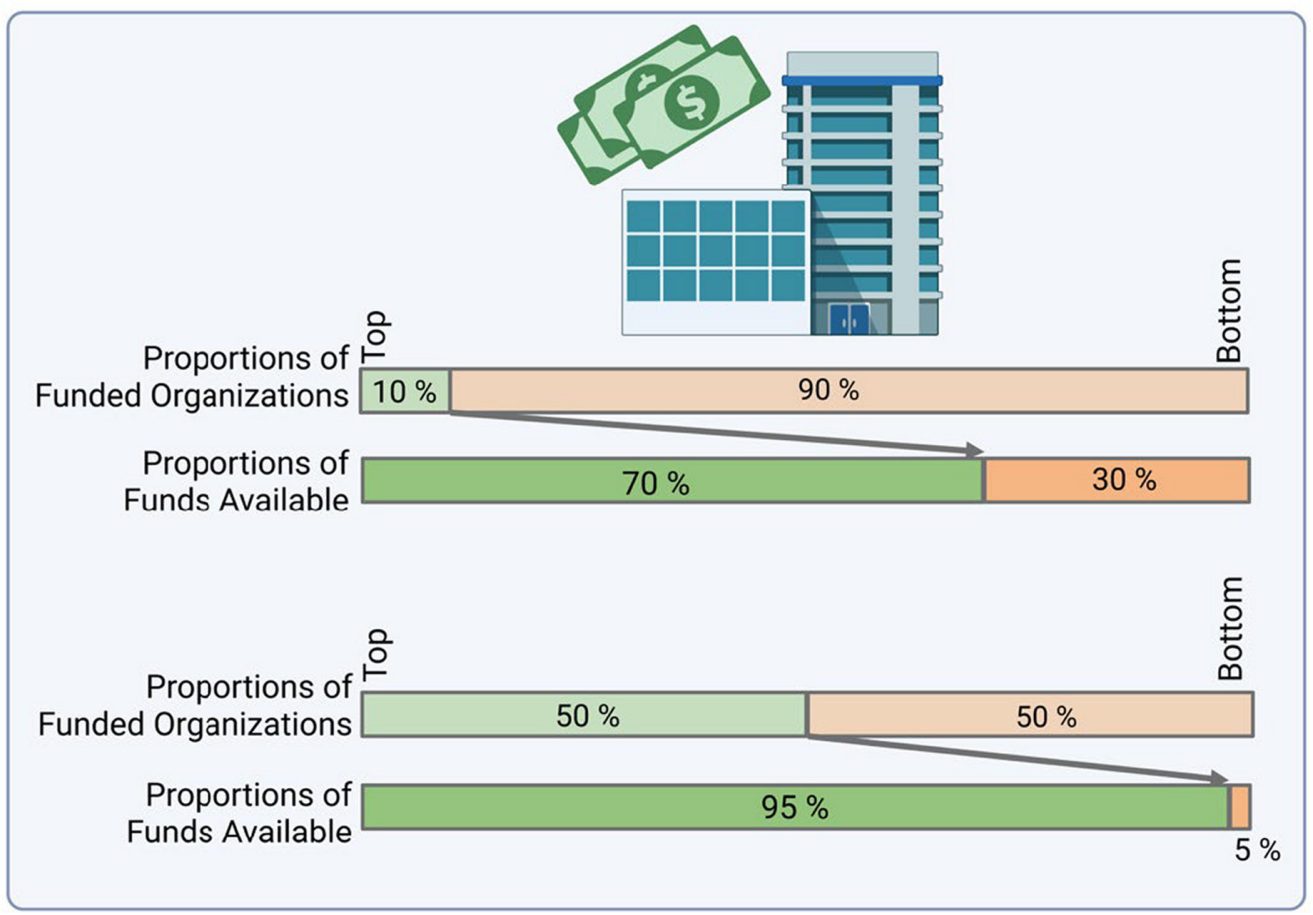
The top decile (10%) of organizations acquired ~70% of NIH funds, whereas the bottom 50% acquired <5% of funds. Adapted from free‐of‐copyright data in Lauer & Roychowdhury (2021). Created in BioRender®.

Be aware that thresholds to achieve retention/tenure/promotion can vary dramatically across institutions. National Institutes of Health (NIH) funding data in the United States reveal that the top decile (10%) of organizations receiving research project grant funding acquired ~70% of the funds available, whereas the bottom 50% of organizations acquired <5% of total funds (Figure 2).^1^ Similarly, the top‐funded centile (1%) of scientists acquired $4.8M as the median in 2020, whereas the remaining 99% of investigators acquired a median of $0.4M.^1^ Therefore, try to identify the goalposts for retention/tenure/promotion—even if no formal thresholds for papers and grants are listed in the faculty handbook. In addition to this two‐part series, there are other resources to help faculty members flourish (e.g., see 2, 3, 4, 5). Use our advice and these resources to build a plan to address as many of the institutional criteria for success as possible. Next, ask yourself if these goals are realistic and achievable, given all your other responsibilities (e.g., family, childcare, sick parents).

## 2 A FRANK EVALUATION OF YOURSELF AND THE INSTITUTION

From the institution's webpages and search engines, you can figure out which faculty members perform research in the area closest to yours and could serve as potential collaborators. You can also calculate from the faculty webpages what fraction of senior faculty members are tenured and/or full professors. Many faculty members post accomplishments on faculty webpages, laboratory websites, or social media. During your search, evaluate if the webpages are designed clearly and contain relevant information on scholarship and teaching. Look especially closely at webpages of newly hired faculty members.

Even if there are no faculty members in your specific area of expertise at the institution, ask yourself if your type of research can be published in the same quartile (e.g., top 25%) of journals. The quartile rankings of journals can be viewed on websites such as *Scopus* (https://www.scopus.com/sources). PubMed can also be leveraged to identify publications by authors affiliated with a specific institution or department (https://pubmed.ncbi.nlm.nih.gov). The reputation of a venerable journal (e.g., an official Society journal) is more meaningful than impact factors, but a candidate's track record is often judged through the lens of impact factors and quartile rankings—rather than the quality of the work itself. Unfortunately, you cannot assume the hiring committees will “read your papers.”

Use public data to calculate approximately how much various faculty members are awarded per year. This type of information is published by the National Institutes of Health (e.g., https://reporter.nih.gov), the National Science Foundation (e.g., https://www.nsf.gov/awardsearch/), the Department of Defense (e.g., https://publicaccess.dtic.mil/search/#/grants/advancedSearch and https://cdmrp.health.mil/search.aspx), and private foundations (https://proposalcentral.com). Bear in mind that these numbers vary depending on degree of seniority, field of research, teaching workload, administrative duties, and the type of institution.

## 3 PRACTICE YOUR JOB TALK WITH CRITICAL THINKERS

You have applied to an institution that is appropriately scaled for your type of work and you were successful at landing an interview request. What next? You need to practice your job talk to tell a good story, and you need to time yourself for a few runs. Note that “less is more” when it comes to a job talk. It may be perceived as rude to go over the allotted time or pack in irrelevant data slides that are not part of your narrative. This can also give the impression that you will overwhelm students when you start to teach in the classroom or mentor the next generation of scientists in the laboratory.

Always choose critical thinkers outside your circle of friends as your test audience when practicing your job talk. The “gruffest” people in the department may be the most helpful. They need to criticize your talk and probe your depth of scientific knowledge, without being worried about what you or others think.

## 4 SCHEDULE THE INTERVIEW

Your job talk is interesting, succinct, and ready for delivery. What next? Schedule the interview without agreeing to a flight that will arrive late and impact your sleep. Avoid a dinner engagement immediately after your arrival, especially if it is a long route with connecting flights. Give yourself time to unwind the night before your interview and ask to present your job talk at your cognitive peak (~10 a.m. to noon for most).

## 5 BRING A LIST OF IMPORTANT QUESTIONS

During the interview, you may leave the impression you are not serious about the position if you fail to pose “good” questions. You could start by asking the chair and dean for their criteria for success in scholarship and teaching. Nebulous or inconsistent criteria are a red flag. Remember that you are interviewing them as much as they are interviewing you. Is there a well‐defined review process to guide faculty toward promotion? Is there a formal mentoring plan that includes extramural scientists in your field? What opportunities are there for collaborations? Are there core facilities, or will you have to work with neighboring institutions with better infrastructure?

If your position involves teaching, find out from the chairperson how large average class sizes are, how many contact/credit hours faculty teach, and how much this number drops and rises over time. Are the number of teaching contact hours subject to buyout upon acquiring sizable funding? Will there be any formal training (or informal mentoring) to succeed in teaching?

If you meet with a representative from Human Resources, ask for the rate of employee turnover. Low faculty or staff retention can be another warning sign. While you are meeting with Human Resources, pay attention to financial perks that dramatically impact the value of your compensation package, such as health insurance and education or retirement benefits. It is also appropriate to ask if relocation costs will be covered.

## 6 OBSERVE DEPARTMENTAL DYNAMICS WITHOUT GOSSIPING

During the interview, quietly observe the nature of the interactions between faculty members. Do they speak collegially? Try to understand why they have not yet left the institution if they have legitimate gripes, while being aware that you do not know the full story. Nonetheless, look for consistency across faculty members in the nature of complaints.

You can also ask to meet with graduate students and other trainees, who can offer unique and unfiltered perspectives about the department. Bear in mind that students and other trainees are often asked their opinions about potential faculty recruits. It is therefore important not to let your guard down, even during casual chatter. Everyone is watching and forming impressions, whether or not they (and you) are conscious of it.

## 7 AFTER THE INTERVIEW

Shortly after the interview, follow up with an email that includes something interesting besides a commonplace thank‐you. If you are writing grants and have one or more under review, mention this. You will be more credible as a researcher if you have recently submitted proposals. You could also follow up with any progress in publications. The more the hiring committee hears positive news, the more they will remember you as unique and successful.

## 8 BEFORE YOU NEGOTIATE THE CONTRACT

You receive notification of the offer, and you decide to accept. What next? You must diplomatically negotiate your contract and the size of your startup package. Your requests will need to be reasonable within the financial context of the new institution (e.g., R1 vs. R2) and department. It is therefore appropriate to ask what size of startup packages have been awarded in recent years.

Make sure that your itemized budget addresses all costs, including, for example, travel to conferences. Salary costs and frequent purchases of disposable items, such as for cell culture, can make your startup dwindle quickly. However, many items can be purchased in bulk with steep “New Lab Discounts” from manufacturers. Sales representatives will be eager to get you hooked on their products early on. Request virtually all that you need up front, because it will not be easy to raise the startup amount, once you submit your request.

Calculate how much space and other resources will be available for your research laboratory, including in the vivarium. Try to acquire a complete list of core facility or shared equipment. Ask if each piece is fully operational. You can then prepare a better itemized budget. Make your equipment list with specifications, model name, and manufacturer, and request manufacturer quotes as if you will buy the equipment within the next 6 months. Let the salesperson know that you are shopping around for the best deal, to ensure competitive pricing.

Aside from negotiating for a technician and trainees to assist you with setting up the lab, you will need to know if graduate students are supported by teaching assistantships, in which case you pay less for them, or research assistantships, in which case you pay the stipend and perhaps the tuition out of your grants or startup. Determine how long the teaching assistantships last and how many years the average student needs to acquire their degree at that institution. You must plan these costs into your budget.

Ask for the duration of your startup account and insist on confirmation in writing, as verbal commitments are not legally binding. Tactfully request a follow‐up email if promises are only made orally. You can also ask for your teaching load to be defined in your appointment letter, at least for the first few years.

Here, we have provided advice on acquiring a faculty or researcher position at an R1/R2 institution. Next, we will help you hit the ground running upon arrival and prepare for an effective retention or promotion package in Part II of this editorial series.

## CONFLICT OF INTEREST STATEMENT

The authors declare no conflicts of interest. No approvals were gathered for this work.

## Data Availability

No data were collected during the creation of this report, but any further questions can be emailed to leakr@duq.edu.
